# Supplementary material for: The Interplay of Epigenetic, Genetic, and Traditional Risk Factors on Blood Pressure: Findings from the Health and Retirement Study
Source: Genes (Basel). 2022 Oct 27;13(11):1959. doi: 10.3390/genes13111959 (PMC9689874; doi:10.3390/genes13111959)
Supplement: Supplementary file 1 [file genes-13-01959-s001.zip › genes-1892878-supplementary.pdf]

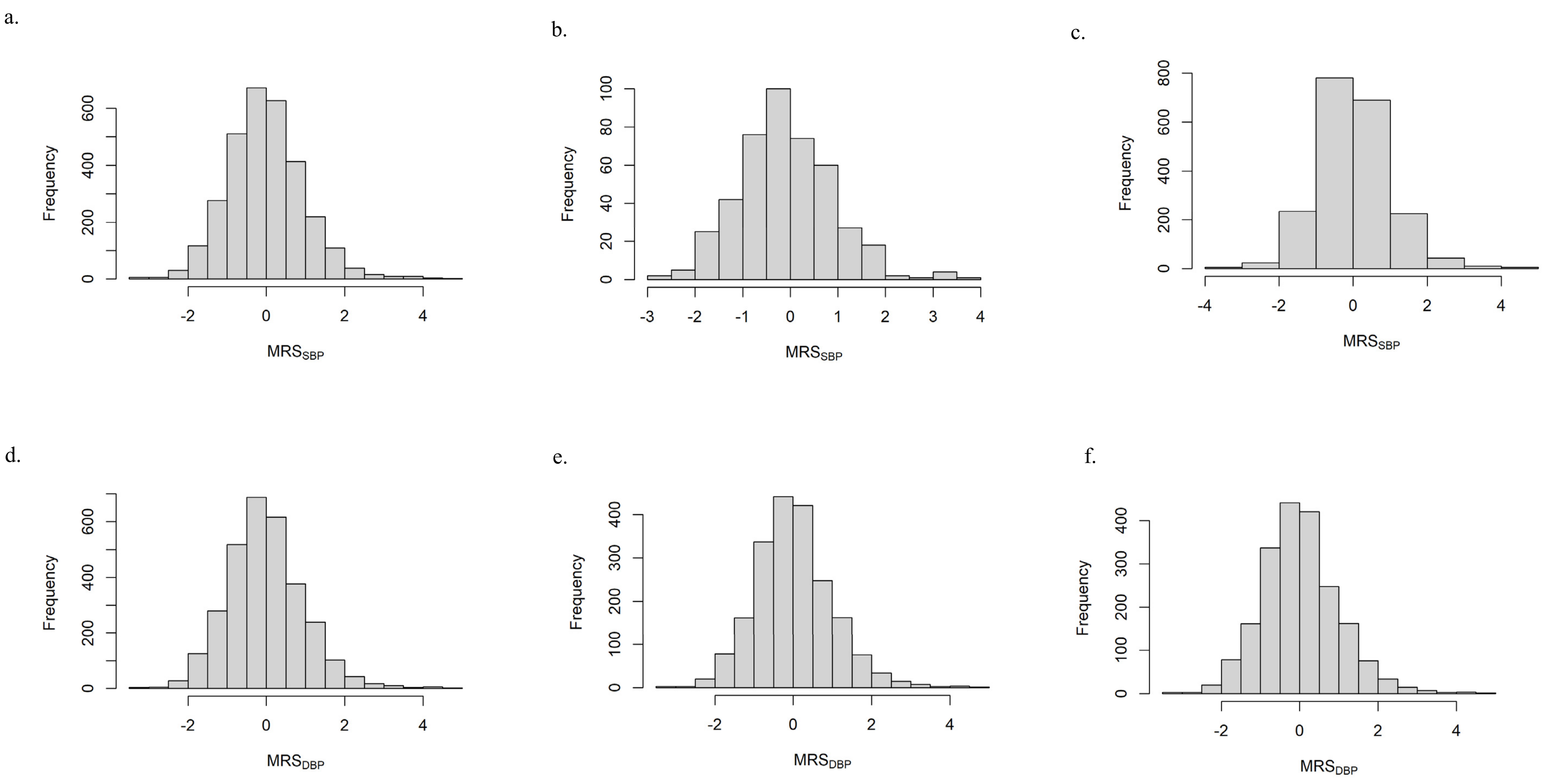

Figure S1. Distribution of systolic (a, b, c) and diastolic (d, e, f) methylation risk scores in the full, African ancestry, and European ancestry samples in the Health and Retirement Study

Table S1. Associations between 13 blood pressure-associated CpGs and systolic blood pressure (SBP) in the African and European ancestry samples of the Health and Retirement Study

		African ancestry						European ancestry					
		Model 1 (*n* = 437)			Model 2 (*n* = 355)			Model 1 (*n* = 2021)			Model 2 (*n* = 1813)		
**CpG site**	**UCSC Gene and location** [b]	**β**	**SE**	***p***	**β**	**SE**	***p***	**β**	**SE**	**P**	**β**	**SE**	***p***
cg23999170	*TSPAN2* (Body)	-0.28	0.18	0.12	-0.22	0.19	0.27	0.01	0.08	0.85	0.02	0.08	0.83
cg16246545	*PHGDH* (Body)	-0.28	0.18	0.13	-0.14	0.2	0.48	-0.12	0.08	0.11	-0.07	0.08	0.34
cg14476101	*PHGDH* (Body)	-0.23	0.14	0.10	-0.04	0.15	0.81	-0.09	0.06	0.15	-0.04	0.06	0.53
cg19693031	*TXNIP* (3'UTR)	0	0.17	1.0	0.09	0.19	0.64	**-0.32**	**0.07**	**1.1×10^{-5} [a]**	**-0.21**	**0.08**	**0.01**
cg08035323	–	-0.05	0.15	0.74	-0.11	0.17	0.52	-0.08	0.06	0.23	-0.11	0.07	0.19
cg06690548	*SLC7A11* (Body)	-0.28	0.22	0.2	-0.15	0.25	0.55	**-0.27**	**0.07**	**4.1×10^{-4} [a]**	**-0.17**	**0.08**	**0.04**
cg18120259	*LOC100132354* (Body)	**-0.56**	**0.26**	**0.03**	-0.54	0.29	0.06	**-0.27**	**0.11**	**0.01**	-0.09	0.11	0.44
cg00533891	*ZMIZ1* (5'UTR)	**-0.33**	**0.17**	**0.05**	-0.34	0.18	0.06	-0.09	0.08	0.26	-0.13	0.08	0.13
cg17061862	–	-0.17	0.15	0.26	-0.12	0.16	0.46	**-0.15**	**0.07**	**0.03**	**-0.14**	**0.07**	**0.04**
cg00574958	*CPT1A* (5'UTR)	-0.61	0.51	0.24	-0.38	0.57	0.50	**-1.24**	**0.28**	**1.1×10^{-5} [a]**	**-0.64**	**0.29**	**0.03**
cg10601624	–	0.03	0.24	0.91	-0.09	0.27	0.74	-0.13	0.11	0.25	-0.16	0.12	0.18
cg22304262	*SLC1A5* (5'UTR; Body)	-0.13	0.21	0.56	0.07	0.24	0.76	**-0.23**	**0.09**	**0.01**	-0.06	0.09	0.48
cg02711608	*SLC1A5* (1st Exon; 5'UTR; Body)	-0.26	0.25	0.30	0.01	0.27	0.96	**-0.55**	**0.14**	**1.2×10^{-4} [a]**	**-0.35**	**0.15**	**0.02**

SE, standard error; Chr, chromosome; SBP, systolic blood pressure. Model 1: SBP ~ CpG sites + age + sex + 10 PCs. Model 2: SBP ~ Model 1 covariates + smoking status + alcohol consumption + BMI + exercise + type 2 diabetes + educational attainment + parental education. Effect sizes (β) correspond to the change SBP (mmHg) associated with a 1% increase in DNA methylation beta value of the CpG. Associations significant at $p < 0.05$ are shown in bold. [a] Significant after Bonferroni correction for 13 tests ($p < 0.0038$). [b] From Illumina annotation as reported in Richard, et al. Am J Hum Genet. 2017;101(6):888-902.

Table S2. Associations between 13 blood pressure-associated CpGs and diastolic blood pressure (DBP) in the African and European ancestry samples the Health and Retirement Study

		African ancestry						European ancestry					
		Model 1 (*n* = 437)			Model 2 (*n* = 355)			Model 1 (*n* = 2021)			Model 2 (*n* = 1813)		
**CpG site**	**UCSC Gene and location [b]**	**β**	**SE**	***p***	**β**	**SE**	***p***	**β**	**SE**	**P**	**β**	**SE**	***p***
cg23999170	*TSPAN2* (Body)	-0.20	0.10	0.05	-0.17	0.11	0.13	0	0.04	0.94	-0.01	0.05	0.76
cg16246545	*PHGDH* (Body)	-0.19	0.10	0.07	-0.11	0.11	0.36	**-0.09**	**0.04**	**0.04**	-0.06	0.05	0.22
cg14476101	*PHGDH* (Body)	**-0.20**	**0.08**	**0.01**	-0.07	0.09	0.39	**-0.07**	**0.04**	**0.04**	-0.04	0.04	0.29
cg19693031	*TXNIP* (3'UTR)	-0.06	0.09	0.49	-0.04	0.11	0.71	-0.08	0.04	0.06	-0.03	0.04	0.45
cg08035323	–	0.01	0.09	0.88	-0.03	0.1	0.8	**-0.07**	**0.04**	**0.05**	-0.07	0.04	0.09
cg06690548	*SLC7A11* (Body)	-0.19	0.13	0.14	-0.13	0.14	0.35	**-0.18**	**0.04**	**$3.1{\times}10^{-5}$ [a]**	**-0.11**	**0.05**	**0.02**
cg18120259	*LOC100132354* (Body)	-0.21	0.15	0.16	-0.27	0.16	0.10	**-0.23**	**0.06**	**$2.3{\times}10^{-4}$ [a]**	**-0.14**	**0.07**	**0.03**
cg00533891	*ZMIZ1* (5'UTR)	**-0.30**	**0.09**	**$1.0{\times}10^{-3}$**	**-0.24**	**0.1**	**0.02**	**-0.1**	**0.05**	**0.03**	**-0.11**	**0.05**	**0.02**
cg17061862	–	-0.13	0.08	0.12	-0.09	0.09	0.33	**-0.09**	**0.04**	**0.02**	**-0.09**	**0.04**	**0.03**
cg00574958	*CPT1A* (5'UTR)	-0.25	0.29	0.38	-0.14	0.33	0.66	**-0.71**	**0.16**	**$9.3{\times}10^{-6}$ [a]**	-0.30	0.17	0.07
cg10601624	–	-0.04	0.13	0.76	-0.12	0.15	0.44	-0.09	0.07	0.19	-0.07	0.07	0.30
cg22304262	*SLC1A5* (5'UTR; Body)	-0.14	0.12	0.26	-0.06	0.14	0.64	**-0.18**	**0.05**	**$3.0{\times}10^{-4}$ [a]**	**-0.11**	**0.05**	**0.04**
cg02711608	*SLC1A5* (1st Exon; 5'UTR; Body)	-0.16	0.14	0.27	0	0.16	0.99	**-0.39**	**0.08**	**$1.8{\times}10^{-6}$ [a]**	**-0.28**	**0.09**	**$1.0{\times}10^{-3}$ [a]**

SE, standard error; Chr, chromosome; DBP, diastolic blood pressure. Model 1: DBP ~ CpG sites + age + sex + 10 PCs. Model 2: DBP ~ Model 1 covariates + smoking status + alcohol consumption + BMI + exercise + type 2 diabetes + educational attainment + parental education. Effect sizes (β) correspond to the change DBP (mmHg) associated with a 1% increase in DNA methylation beta value of the CpG. Associations significant at $p < 0.05$ are shown in bold. [a] Significant after Bonferroni correction for 13 tests ($p < 0.0038$). [b] From Illumina annotation as reported in Richard, et al. Am J Hum Genet. 2017;101(6):888-902.

Table S3. Associations between genetic risk scores and blood pressure by ancestry in the Health and Retirement Study

	Systolic blood pressure								Diastolic blood pressure							
	Model 1				Model 2				Model 1				Model 2			
	n	**β**	**SE**	***p***	***n***	**β**	**SE**	***p***	***n***	**β**	**SE**	***p***	***n***	**β**	**SE**	***p***
African ancestry	437	-1.93	2.48	0.44	355	-3.55	2.64	0.18	437	-0.31	1.22	0.800	355	-0.86	1.31	0.515
European ancestry	**2021**	**5.03**	**0.58**	$\mathbf{1.2\times10^{-17}}$	**1813**	**4.84**	**0.60**	$\mathbf{9.3\times10^{-16}}$	**2021**	**2.83**	**0.33**	$\mathbf{2.4\times10^{-17}}$	**1813**	**2.82**	**0.34**	$\mathbf{3.6\times10^{-16}}$

SE, standard error; SBP, systolic blood pressure; DBP, diastolic blood pressure. Model 1: SBP/DBP ~ GRS_{SBP}/GRS_{DBP} + age + sex + 10 PCs. Model 2: Model 1 covariates + smoking status + alcohol consumption + BMI + exercise + type 2 diabetes + educational attainment + parental education. Effect sizes (β) correspond to the change in SBP/DBP (mmHg) associated with a 1 standard deviation increase in the GRS. Associations significant at $p < 0.05$ are shown in bold.

Table S4. Sensitivity analysis using sampling weights to assess the associations between methylation risk scores and blood pressure in the full sample and by ancestry in the Health and Retirement Study

	Systolic blood pressure								Diastolic blood pressure							
	Model 1				Model 2				Model 1				Model 2			
	**β**	**SE**	***p***	***n***	**β**	**SE**	***p***	***n***	**β**	**SE**	***p***	***n***	**β**	**SE**	***p***	***n***
Full sample	**2.69**	**0.56**	**$1.7×10^{-5}$**	2970	**2.15**	**0.58**	**$7.4×10^{-4}$**	2607	**1.93**	**0.32**	**$2.9×10^{-7}$**	2970	**1.63**	**0.36**	**$7.4×10^{-5}$**	2607
African ancestry	2.24	1.15	0.06	420	1.33	0.98	0.20	342	**1.83**	**0.72**	**0.016**	420	1.36	0.79	0.11	342
European ancestry	**2.59**	**0.62**	**$1.5×10^{-4}$**	1977	**2.13**	**0.65**	**$2.4×10^{-3}$**	1772	**1.91**	**0.35**	**$3.0×10^{-6}$**	1976	**1.57**	**0.40**	**$4.5×10^{-4}$**	1711

SE, standard error; MRS, methylation risk score; SBP, systolic blood pressure; DBP, diastolic blood pressure. Model 1: SBP/DBP ~ MRS_{SBP}/MRS_{DBP} + age + sex + 10 PCs. Model 2: Model 1 covariates + smoking status + alcohol consumption + BMI + exercise + type 2 diabetes + educational attainment + parental education. Effect sizes (β) correspond to the change in SBP/DBP (mmHg) associated with a 1 standard deviation increase in the MRS. Associations significant at $p < 0.05$ are shown in bold

Table S5. Sensitivity analysis using sampling weights to assess the of associations between methylation and genetic risk scores and blood pressure by ancestry in the Health and Retirement Study

	Systolic Blood Pressure			Diastolic Blood Pressure		
**Sample/Predictor**	**β**	**SE**	***p***	**β**	**SE**	***p***
African ancestry (N=342)						
MRS	1.19	0.94	0.23	1.27	0.77	0.12
GRS	-3.19	2.46	0.21	-1.64	1.56	0.31
European ancestry (N=1772 for SBP and 1771 for DBP)						
MRS	**1.99**	**0.63**	**3.6×10^{-3}**	**1.44**	**0.39**	**7.5×10^{-3}**
GRS	**4.65**	**0.54**	**1.1×10^{-9}**	**2.70**	**0.36**	**1.4×10^{-8}**

MRS, methylation risk score; GRS, genetic risk score; SBP, systolic blood pressure; DBP, diastolic blood pressure. Model: SBP/DBP ~ MRS_{SBP}/MRS_{DBP} + GRS_{SBP}/GRS_{DBP} + age + sex + 10 PCs + smoking status + alcohol consumption + BMI + exercise + type 2 diabetes + educational attainment + parental education. MRS effect sizes (β) correspond to the change in SBP/DBP (mmHg) associated with a 1 standard deviation increase in the MRS. GRS effect sizes (β) correspond to the change in SBP/DBP (mmHg) associated with a 1 standard deviation increased in the GRS. Associations significant at $p < 0.05$ are shown in bold

Table S6. Sensitivity analysis using sampling weights to assess the interaction analysis between blood pressure methylation risk scores and risk factors by ancestry in the Health and Retirement Study.

		Systolic blood pressure							Diastolic blood pressure						
**Multiplicative interaction term evaluated**	**Sample**	***n***	**β_{MRS}**	**p_{MRS}**	**β_{RF}**	**p_{RF}**	**$\beta_{interaction}$**	**$p_{interaction}$**	***n***	**β_{MRS}**	**p_{MRS}**	**β_{RF}**	**p_{RF}**	**$\beta_{interaction}$**	**$p_{interaction}$**
MRS × GRS	AA	342	1.26	0.19	-3.59	0.17	**-2.36**	**0.03**	342	1.32	0.10	-1.82	0.27	-0.81	0.33
	EA	1772	**1.98**	**3.8×10^{-3}**	**4.65**	**1.5×10^{-9}**	-0.19	0.72	1771	**1.46**	**7.3×10^{-4}**	**2.70**	**1.4×10^{-8}**	-0.46	0.18
MRS × age [a]	Full sample	2607	**2.00**	**1.0×10^{-3}**	**4.63**	**2.4×10^{-8}**	**-1.48**	**5.2×10^{-3}**	2607	**1.51**	**4.7×10^{-5}**	-0.67	0.06	**-1.09**	**1.6×10^{-3}**
	AA	342	1.67	0.16	0.76	0.65	1.04	0.37	342	1.33	0.14	**-2.19**	**0.02**	-0.08	0.91
	EA	1772	**2.11**	**2.1×10^{-3}**	**4.60**	**3.4×10^{-8}**	**-1.82**	**4.9×10^{-3}**	1771	**1.53**	**2.2×10^{-4}**	-0.59	0.13	**-1.25**	**2.0×10^{-3}**
MRS × sex [b]	Full sample	2607	**2.37**	**3.0×10^{-3}**	**-2.95**	**6.5×10^{-3}**	-0.45	0.62	2607	**1.66**	**2.6×10^{-3}**	-0.02	0.98	-0.06	0.92
	AA	342	-1.05	0.40	-1.72	0.47	4.50	0.06	342	-0.17	0.88	-1.14	0.44	2.91	0.05
	EA	1772	**2.19**	**1.0×10^{-2}**	**-2.65**	**0.02**	-0.12	0.89	1771	**1.45**	**0.01**	0.65	0.37	0.23	0.72
MRS × high school degree or equivalent [c]	Full sample	2607	-0.11	0.93	**-3.98**	**0.02**	2.47	0.09	2607	0.42	0.61	-0.87	0.31	1.31	0.16
	AA	342	-0.37	0.86	-5.02	0.19	2.21	0.39	342	1.75	0.43	-0.46	0.84	-0.33	0.89
	EA	1772	-0.34	0.84	**-4.95**	**0.02**	2.59	0.20	1771	0.69	0.46	-0.83	0.48	0.91	0.40
MRS × at least some college [d]	Full sample	2607	**1.58**	**0.03**	-1.38	0.13	0.93	0.21	2607	0.85	0.09	-0.72	0.18	**1.27**	**0.03**
	AA	342	-0.41	0.68	-2.48	0.35	3.36	0.08	342	0.17	0.84	-1.21	0.39	2.47	0.13
	EA	1772	**2.04**	**0.04**	-1.03	0.32	0.11	0.91	1771	1.21	0.06	-0.45	0.47	0.56	0.41

AA, African ancestry; EA, European ancestry; MRS, methylation risk score; GRS, genetic risk score; RF, risk factor; SBP, systolic blood pressure; DBP, diastolic blood pressure. Model: DBP/SBP ~ corresponding MRS/GRS + age + sex + 10 PCs + smoking status + alcohol consumption + BMI + exercise + type 2 diabetes + educational attainment + parental education + corresponding multiplicative interaction term (MRS × GRS/age/sex/high school degree/at least some college education). MRS effect sizes (β_{MRS}) correspond to the change in SBP/DBP associated with a 1 standard deviation increase in the MRS. Risk factor effect sizes (β_{RF}) correspond to the change in SBP/DBP associated with a 1 unit increase in the risk factor for continuous measures or at the non-reference level for categorical variables. Interaction effect sizes ($\beta_{interaction}$) correspond to the change in effect of β_{MRS} on SBP/DBP for each 1 unit increase (or level) of the risk factor. Associations significant at $p < 0.05$ are shown in bold. [a] Age was centered and scaled. [b] Reference group = male sex. [c] Reference group = less than high school degree. [d] Reference group = no college education.
